# Ferroptosis-Related Flavoproteins: Their Function and Stability

**DOI:** 10.3390/ijms22010430

**Published:** 2021-01-04

**Authors:** R. Martin Vabulas

**Affiliations:** Charité-Universitätsmedizin, Institute of Biochemistry, Charitéplatz 1, 10117 Berlin, Germany; martin.vabulas@charite.de; Tel.: +49-30-4505-28176

**Keywords:** flavoproteins, riboflavin, ferroptosis, lipid peroxidation, protein quality control

## Abstract

Ferroptosis has been described recently as an iron-dependent cell death driven by peroxidation of membrane lipids. It is involved in the pathogenesis of a number of diverse diseases. From the other side, the induction of ferroptosis can be used to kill tumor cells as a novel therapeutic approach. Because of the broad clinical relevance, a comprehensive understanding of the ferroptosis-controlling protein network is necessary. Noteworthy, several proteins from this network are flavoenzymes. This review is an attempt to present the ferroptosis-related flavoproteins in light of their involvement in anti-ferroptotic and pro-ferroptotic roles. When available, the data on the structural stability of mutants and cofactor-free apoenzymes are discussed. The stability of the flavoproteins could be an important component of the cellular death processes.

## 1. Introduction

Human flavoproteome encompasses slightly more than one hundred enzymes that participate in a number of key metabolic pathways. The chemical versatility of flavoproteins relies on the associated cofactors, flavin mononucleotide (FMN) and flavin adenine dinucleotide (FAD). In humans, flavin cofactors are biosynthesized from a precursor riboflavin that has to be supplied with food. To underline its nutritional essentiality, riboflavin is called vitamin B2.

In compliance with manifold cellular demands, flavoproteins have been accommodated to operate at different subcellular locations [1]. The largest fraction of flavoproteins can be found in mitochondria, which is not surprising, given the central metabolic role of this organelle. Flavoproteins reside in all subcompartments of a mitochondrium, including its membranes. One example of a mitochondrial membrane flavoprotein is NDUFV1, the core subunit of the respiratory chain Complex I, associated with FMN that transfers electrons from NADH to the Fe-S clusters [2]. Another prominent resident of mitochondrial membranes is the FAD-bound catalytic subunit of succinate dehydrogenase (Complex II) [3]. Noteworthy, the apoptosis-inducing flavoprotein AIF1 is also anchored in the inner mitochondrial membrane [4]. In addition, transmembrane and membrane-associated flavoproteins can be found also outside mitochondria, for example, at the plasma membrane and in the endoplasmic reticulum. There have been recent advances in untangling the cellular complexity of flavoproteome using structure-based evolutionary analysis [5].

One of the reasons for the association of flavoproteins with cellular membranes is their involvement in lipid biochemistry. This activity brings flavoproteins into the spotlight of the cellular death pathways. The membrane lipid reduction and oxidation (redox) reactions, specifically phospholipid peroxidation, has recently become a focus of intense research following the discovery of ferroptosis, an iron-dependent non-apoptotic cell death [6,7]. Ferroptosis is driven by peroxidation of polyunsaturated fatty acids (PUFAs) when the glutathione-based repair of oxidative damage becomes insufficient [8,9]. Excitingly, it has become clear that the lipid damage-related processes represent a significant pathogenesis component of diverse pathologies [10]. Flavoproteome is involved prominently in these processes. Consequently, the detailed understanding of the anti-ferroptotic and pro-ferroptotic roles played by flavoproteins is necessary in order to develop novel therapeutic approaches. The manipulation of the structural stability of flavoproteins is one of the possibilities to adjust their function and thus deserves thorough consideration.

## 2. Human Flavoproteome

Human flavoproteins constitute an important component of the cellular metabolic network [1]. The enzymatic roles of flavoproteins are determined by the associated flavin cofactors FMN and FAD. The majority of human flavoproteins contain FAD, but only a few are associated with FMN. Very few proteins have both cofactors, for example, cytochrome P450 oxidoreductase (POR) and all three paralogs of nitric oxide synthase. In several cases, the isoalloxazine ring of the flavin cofactor is covalently linked to a histidine or a cysteine residue of the protein. One example of a covalently attached FAD is succinante dehydrogenase in mitochondria. Biochemical reasons for the covalent association are not completely clear, but might include the saturation of the active site, the adjustment of the redox potential, facilitation of reactivity and protein stabilization. From the structural perspective, Rossmann fold is the most prevalent fold among human FAD-binding proteins, closely followed by the Acyl-CoA dehydrogenase fold [1]. Most human FMN-binding proteins have Flavoprotein and TIM barrel folds. In FAD-containing proteins, the pyrophosphate binds to the most strongly conserved sequence motifs, suggesting that pyrophosphate binding is important for molecular recognition of the cofactor [11].

Flavoenzymes catalyze a large variety of diverse reactions. The tricyclic isoalloxazine ring system of flavin cofactors provides necessary redox versatility, explaining the metabolic importance of the flavoproteome [12]. Both one-electron and two-electron transfer are possible, thermodynamically and kinetically; therefore, the one-electron reduced flavin semiquinone and the two-electron reduced dihydroflavin both are biologically relevant. This is in contrast to the obligate two-electron transferring NAD(P)H and one-electron transferring iron. Mechanistically, flavoenzymes generate and react with carbanions, radicals and hydrides [12]. During reduction in the flavin cofactor, the substrates are typically oxidized by transfer of two electrons. Reoxidation of reduced flavin can happen as a reverse reaction. In cases like this, hydride transfer would take place, representing the two-electron at a time reaction. Additionally, the one-electron oxidation of the flavin cofactor can happen; for example, one electron transfer from flavin to the iron-sulfur cluster.

Human flavoenzymes are typically intracellular proteins. There are only a couple of exceptions. For example, renalase is an FAD-dependent amine oxidase that is secreted into the blood from the kidney. Its activity decreases cardiac contractility and rate. Catecholamines promote synthesis and secretion of renalase [13]. Another example is quiescin sulfhydryl oxidases (QXOSs). These Golgi apparatus-localized flavoenzymes are disulfide catalysts and can be secreted. QSOX1 activity was required for the incorporation of laminin into the extracellular matrix; if synthesized without QSOX1, the matrix could not support cell adhesion [14]. More than half of the intracellular flavoproteins are localized in mitochondria and peroxysomes; two organelles with key functions in cellular metabolism. As mentioned above, many flavoproteins associate with cellular membranes (Table 1).

## 3. Ferroptosis

**Figure 1:**
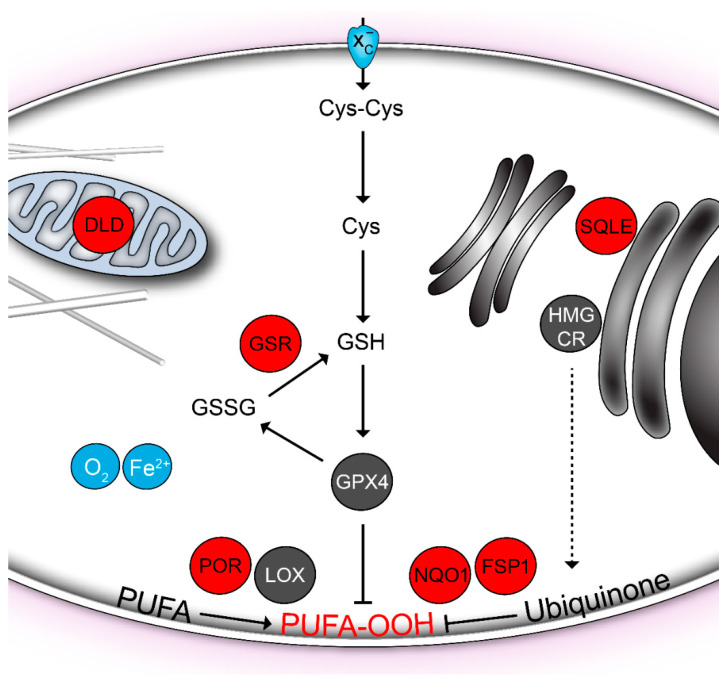
Ferroptosis-related flavoproteins (red). Lipid hydroperoxides (PUFA-OOH) in cellular membranes are generated during aerobic metabolism by the lipoxygenases (LOX) and cytochrome P450 oxidoreductase (POR). Molecular oxygen (O_2_) and iron ions (Fe^2+^) are required for lipid peroxidation. Hydroperoxides are reduced by the glutathione peroxidase GPX4 in the presence of glutathione (GSH). A precursor of glutathione, the cystine (Cys-Cys), is supplied by the transporter System x_c_^-^. Glutathione reductase (GSR) regenerates oxidized glutathione (GSSG) back to its reduced state. In addition, the endogenous lipophilic anti-oxidant ubiquinone can reduce the oxidative lipid damage. It is regenerated by the reducing enzymes NQO1 and FSP1. Ubiquinone is biosynthesized in the mevalonate pathway, which is critically controlled by the HMG-CoA reductase (HMGCR). Another mevalonate pathway enzyme, the squalene monooxygenase (SQLE), is downstream of the branching point towards ubiquinone. Nevertheless, it is also involved in the ferroptosis control [16]. The mitochondrial dihydrolipoamide dehydrogenase (DLD) has been recently found to be required to induce ferroptosis upon cystine deprivation [17].

Ferroptosis is a type of eukaryotic cell death characterized by the iron-dependent accumulation of lipid hydroperoxides to lethal levels [10]. Ferroptosis depends on intracellular iron and is morphologically and biochemically distinct from other forms of cell death, such as apoptosis and necrosis [7]. The antioxidative glutathione system is critical in protecting against ferroptosis (Figure 1). Here, glutathione-dependent glutathione peroxidase 4 (GPX4) is able to restrict lipid peroxidation [8,9], which is unavoidable during aerobic metabolism. To explain the importance of GPX4, it is assumed that the hydroperoxides are continuously reduced by GPX4 in the presence of glutathione [15]. In the glutathione system, flavoprotein glutathione reductase is responsible for sustaining sufficient levels of reduced glutathione in the cell. When GPX4 reaction becomes limiting, ferrous iron initiates lipid peroxidation by decomposing the hydroperoxides. The oxidative chain reaction then leads to irreparable lipid damage and cell death.

Lipid oxidation can proceed autocatalytically or enzymatically. Considering the enzymatic pathway, lipoxygenases (LOXs) have been implicated in driving ferroptotic damage of PUFAs [18,19]. Especially LOX15 has been thought to be central in ferroptosis. On the other side, some cell types lacking LOX activity are known to be still susceptible to ferroptotic death. As a possible explanation, it has been shown that some LOX inhibitors operated as radical-trapping antioxidants [20]. The authors concluded that LOX may contribute to the cellular pool of lipid hydroperoxides, but it is the lipid autooxidation that drives ferroptosis. Yet another explanation could be the presence of further drivers of lipid peroxidation in the cell. For example, the flavoprotein cytochrome P450 reductase has been shown recently to oxidize polyunsaturated phospholipids [21].

The endogenous membrane antioxidant ubiquinone (coenzyme Q) is synthesized by the mevalonate pathway. In preventing peroxidative damage to lipids, reduced ubiquinone is as effective as α-tocopherol [22]. Not surprisingly, the inhibition of the upstream HMG-CoA reductase enhanced ferroptosis induction by FIN56 [16]. Ubiquinone cannot be recycled by ascorbate, but it can be reduced by some enzymes; in the case of the flavoprotein NQO1, this activity has been known for a while [23]. In contrast, the ubiquinone-reducing capacity of the flavoprotein AIFM2 has been discovered only recently [24,25,26]. Consequently, AIFM2 turned out to be able to complement the loss of GPX4.

In the following sections, ferroptosis-related human flavoproteins will be discussed in more detail paying particular attention to the available data on structural and functional stability of the proteins.

## 4. GSR

Glutathione reductase (GSR) is a FAD-containing flavoprotein localized in the cytosol. Glutathione is the reducing substrate in the GPX4 reaction and thus it is the key component of the anti-ferroptotic network. Its cellular availability mirrors the interplay of diverse biochemical processes, such as the uptake of the precursor cystine, the GSH synthesis, conjugation, oxidation and reduction. GSR uses NADPH, two essential cysteins and an activated histidine to reduce an oxidized GSSG dimer into two GSH molecules [27]. NADPH and GSSG bind to separate sites on the GSR, and the associated FAD cofactor lying in-between drives a cycle of redox half reactions. Despite its central role in glutathione homeostasis, GSR does not seem to be essential to maintain the reduced glutathione pool in *E. coli* [28]. In contrast, yeast strains deficient in GSR show higher levels of GSSG and are more sensitive to oxidative stress [29]. However, yeast cells remain viable if the thioredoxin system is intact. The recombinant flavoprotein thioredoxin reductase could reduce oxidized glutathione in vitro. The compensatory effects from other parts of the cellular anti-oxidative machinery might explain rather mild consequences of GSR defects also in other species, including humans.

The first pathogenic mutation identified in human GSR was its C-terminal truncation, which manifested in hemolytic crises after eating fava beans and in cataract development [30,31]. The GSR activity was almost completely lacking in the erythrocytes of affected patients and it could not be rescued by riboflavin in vivo or FAD in vitro. Intracellular enzyme levels were very low to undetectable, which indicated the destabilization of the protein structure. Another mutation, G330A, affected a conservative residue in the FAD-binding motif [31]. When the recombinant mutant was tested in vitro, it showed a strongly impaired thermostability. Interestingly, the patient with the G330A variant was found to be a compound heterozygote bearing a nonsense mutation at W287 in another GSR allele. A similar truncation (S216) is found frequently in tumor samples [32]. It remains to be clarified whether the inactivation of GSR by this somatic mutation has a functional relevance during tumorigenesis.

As discussed above, thioredoxin reductases (TXNRDs) can complement the GSR function in regenerating the reduced glutathione pool in the cell. Conceivably, TRXNDs should be able to support GPX4 in its anti-ferroptotic activity. Thus, it came as a surprise that the loss of TXNRD1 protected pancreatic cancer cells from ferroptosis upon GPX4 inhibition [33]. The authors found that the loss of TXNRD1 increased the levels of GPX4 protein by increasing the cellular pool of selenocystein. Selenocystein is a scarce amino acid required for the structure and function of both polypeptides, thus the disappearance of one facilitated the biogenesis of the second. The case is a good example of a complex relationship between protein biogenesis, structure and function when considered in the natural context of limited cellular resources. Conserved structural elements in different flavoproteins support their shared biochemical roles in the cell [5]. How much this structural relationship contributes to the complementary or antagonistic functions of ferroptosis-related flavoproteins remains to be investigated.

## 5. AIFM2 (FSP1)

In humans, apoptosis-inducing factors (AIF)M1-M3 comprise a family of flavoproteins, all of which have been thought to reside in mitochondria and participate in caspase-independent apoptotic cell death. AIFM1 is the most-analyzed member of the group [5,24,34]. Its structure revealed a gluthatione reductase-like fold [5,35], a curious coincidence when considering the anti-ferroptotic function of the AIF family. Mature AIFM1 is anchored in the inner membrane of mitochondria, exposing its C-terminal part to the intermembrane space [4]. Upon apopotosis induction, AIFM1 is proteolytically processed to be released from the membrane and subsequently translocated to the cytosol and the nuclei. The flavoprotein function in the nuclei does not seem to depend on other cytoplasmic factors and results in chromatin fragmentation [36]. It is believed that AIFM1 associates with nucleases, such as endonuclease G and the macrophage migration inhibitory factor, to execute DNA fragmentation. On the other side, it has been reported that a substantial fraction, approximately 30% of the total pool of AIFM1, resides at the outer mitochondrial membrane on the cytosolic side and might be sufficient to cause cell death, even without an input from the intramitochondrial pool [37].

AIFM2 (also known as apoptosis-inducing factor-homologous mitochondrion-associated inducer of death; AMID) shows significant sequence similarity with AIFM1 and both proteins were assumed initially to have similar subcellular localization and pro-apoptotic functions [38]. However, AIFM2 lacks a mitochondrial targeting sequence and instead features an N-terminal myristoylation motif. Inhibitor and mutational studies revealed the importance of myristoylation to target the flavoprotein to lipid droplets and plasma membrane [25]. In support, lipid-droplet localization of AIFM2 has been discovered in an unbiased screen using proximity labeling in combination with mass spectrometry [39]. Another study reported the localization of GFP-fused AIFM2 in an unspecified perinuclear membrane compartment and its partial overlapping with the endoplasmic reticulum and Golgi apparatus markers [26]. In addition to and independent of the involvement in apoptosis, AIF family members were assigned the NAD(P)H oxidase function [40,41]. Interestingly, AIFM2 was found to contain 6-hydroxy-FAD as cofactor [41]. The 6-hydroxy-FAD is a rather exotic flavin variant, which can be found in other flavoproteins, only in low abundance. Its relevance for the AIFM2 function remains to be clarified.

Recently, there have been important advances in understanding the additional roles of AIFs. It was shown that both AIFM1 and AIFM2 can function as NADH dehydrogenases with ubiquinone as a physiological electron acceptor [24]. This work identified AIFs as the elusive candidate dehydrogenases capable of bypassing the respiratory chain Complex I in human cells. These alternative NADH dehydrogenases (NDH-2) have been known for a long time to exist in other species, such as yeast and plants, and they are believed to confer metabolic plasticity to the respective organisms. The discovery of similar activity of human AIFs opened new possibilities to better understand the metabolic adaptation of human cells. Furthermore, the capacity of AIF flavoproteins to reduce the endogenous antioxidant ubiquinone (CoQ) is obviously also relevant in the context of cellular membrane protection from oxidative damage. The one electron-reduced (semiquinone) and two electron-reduced (ubiquinol) forms are at the basis of CoQ function not only as electron carriers during the mitochondrial oxidative respiration, but also as a membrane-resident antioxidant. Thus, it was perfectly reasonable that AIFM2 was very recently shown to suppress the oxidative membrane damage-driven cell death, ferroptosis. Two very different screening strategies were used to uncover additional ferroptosis regulators and both screens converged on AIFM2 [25,26]. To underscore its new function, the protein was renamed ferroptosis suppressor protein 1 (FSP1). Importantly, ubiquinone-reducing activity was shown to be the key for the anti-ferroptotic role of the flavoprotein. Although able to reduce ubiquinone too, AIFM1 was not found among hits with anti-ferroptotic activity. One possible explanation is the different cellular localizations of the two paralogs: AIFM1 is constrained to mitochondria and has limited access to other cellular membranes.

When mutated, AIFM1 can lead to severe mitochondrial diseases. The first pathogenic variant was identified ten years ago in patients presenting with involuntary movements, peripheral motor neuropathy and muscular atrophy [42]. The arginine deletion (R201del) localized in the FAD-binding domain was shown to affect the stability and activity of the flavoprotein. Many more mutations in AIFM1 have been identified meanwhile and some of the mutant proteins have been thoroughly characterized in vitro [43]. These analyses concluded that only a strong decrease in cellular steady-state levels or impairment of the enzymatic activity of AIFM1 lead to early and severe forms of disease. In contrast, less pronounced structural changes manifest in slowly progressing neurodegeneration [43]. What about AIFM2 (FSP1) mutations? Glutamate 156 that is required for the binding FAD was exchanged to alanine (G156A) and the functional consequences analyzed [25]. The mutation did not affect cellular levels and the localization of FSP1, but it impaired the reduction in ubiquinone and the anti-ferroptotic activity of the protein. The effect of naturally occurring mutations, such as M135T and D288N, has not been investigated yet. This question is relevant in regard to the ferroptosis sensitivity, because both these mutations do occur more frequently in tumors [32].

## 6. NQO1

Human NAD(P)H:quinone oxidoreductase (NQO)1 belongs to a family of quinone reductases found across such diverse taxa as bacteria, fungi and archea. There is a highly similar paralog of NQO1 in humans, that is called NQO2. However, a number of organisms contain only one of the two proteins. Mammalian NQO1 was discovered and characterized some 70 years ago [44,45]. At that time, the protein was named DT-diaphorase to underscore its capacity to oxidize equally efficiently both NADH and NADPH, diphosphopyridine nucleotide and triphosphopyridine nucleotide, as they were called then. Using NAD(P)H, NQO1 catalyzes the two-electron reduction in endogenous and exogenous quinones to hydroquinones. The production of semiquinones by one-electron reductases would be much more dangerous for a cell, because the redox cycling of semiquinones in the presence of molecular oxygen results in the formation of reactive oxygen species [46].

One of the remarkable features of NQO1 is its high inducibility by diverse environmental insults. NQO1 expression is driven by the antioxidant response element (ARE) and the xenobiotic response element (XRE) located in its promoter region [47]. Accordingly, the activation of the transcription factors NF-E2 p45-related factor 2 (Nrf2) and the arylhydrocarbon receptor (AhR) must precede the enhanced gene transcription and happens in response to a plethora of chemical and physical stressors, such as polycyclic aromatic hydrocarbons, azo dyes, hydrogen peroxide, ionizing radiation, photodynamic therapy, nanoparticle exposure and shear stress in blood vessels [48]. It is interesting to note that some tumor types show constitutively elevated NQO1 protein levels. For example, high levels of NQO1 were detected in non-small cell lung cancer (adenocarcinoma, squamous cell carcinoma, and bronchoalveolar carcinoma), but not in small cell lung cancer or carcinoid lung tumors [49]. The reason for this specificity remains unclear and tumor-type specific roles of NQO1 could be one possible explanation. One of the roles could be protection from ferroptosis. For example, the knockdown of NQO1 in hepatocellular carcinoma cells increased their vulnerability to this form of cell death [50].

The presence of the flavin cofactor in its hydroquinone form (FADH_2_) in NQO1 allows for the reduction in superoxide. The relevance of this activity remains unclear, though, because the rate constant of reduction is very low, going four orders of magnitude below that of superoxide dismutase [51]. On the other side, high concentration of NQO1 following upregulation during oxidative stress bears a possibility, at least in principle, that NQO1 contributes significantly to the antioxidative protection of cells because of superoxide reduction. This role of NQO1 can become relevant in circumstances when cellular levels of superoxide dismutase are low, such as in some cardiovascular cell types. For example, when lysates of aortic smooth muscle A10 cells or those of cardiac H9c2 cells were tested, they were found to inhibit pyrogallol autooxidation in NQO1- and NAD(P)H-dependent manner [52].

As mentioned above, ubiquinone (CoQ) protects cellular membranes from oxidative damage. The capacity of NQO1 to reduce ubiquinones has been known for quite some time [23]. Beyer at al. demonstrated that the addition of NADH and NQO1 from rat liver to large unilamellar or multilamellar vesicles containing CoQ resulted in its complete reduction. Furthermore, NQO1 in this experimental model was able to protect membrane lipids from peroxidation in the presence of the radical initiator 2,2′-azobis(2,4-dimethylvaleronitrile). Importantly, NQO1 also protected cellular membranes of hepatocytes from loss of permeability under adriamycin treatment and this protection was reversed with dicoumarol, the inhibitor of NQO1 [23]. While considering the biological relevance of NQO1-dependent reduction in ubiquinone, the cellular localization of the enzyme represents an important issue. The cytosol is the main cellular compartment where this flavoprotein can be found. However, other cellular subcompartments, such as organellar and plasma membranes, deserve more thorough scrutiny, especially in regard to the presence of NQO1 upon its massive upregulation during oxidative stress and in some tumor types.

The reducing activity of NQO1 can be lost to a different degree due to structural changes caused by mutations. Proline-to-serine change at position 187 (P187S) is an interesting variant for several reasons. First, the allele is found relatively often, showing the frequency of 25. In some groups, such as East Asian populations, the allele frequency reaches as much as 46%. Second, P187S renders the protein highly unstable. In cells homozygous for the mutation, NQO1 levels were very low or not detectable and the enzymatic activity was lacking [53]. The lack of activity could be explained by the impaired binding of the cofactor FAD to the enzyme [54]. It turned out that the mutation causes structural and dynamic changes affecting, at a distance, the FAD binding site and increasing the flexibility of the C-terminal tail [55,56]. Third, the structural defect in the mutant protein phenocopies the in vitro and in vivo behavior of the cofactor-free wild-type NQO1 [57]. Specifically, wild-type apo-NQO1, similar to the P187S mutant, became unstable in cells, which lead to its increased degradation by the 26S proteasome. The proteasome-targeting ubiquitination of both wild-type apo-NQO1 and P187S mutant was executed by the ubiquitin ligase CHIP (C terminus of HSC70-interacting protein). CHIP recognized its targets directly via the flexible C-terminus of NQO1 because the C-terminal truncation of NQO1 strongly affected the ubiquitination.

It remains to be determined how general the recognition mechanism used by the cellular protein quality control (PQC) machinery to detect cofactor-free flavoproteins is. It is interesting to note here, that the paralog NQO2 naturally lacks the 43 amino acid-long C-terminal tail of NQO1, otherwise showing high structural similarity. The truncation translates into an altered site to bind pyridine nucleotides. As a consequence, NQO2 prefers nicotine riboside over NAD(P)H and differs also in its substrate preferences. The lack of the flexible tail in NQO2 was conceivably the reason why CHIP failed to recognize the flavoprotein regardless of the presence/absence of the cofactor [57]. At the same time, the levels of NQO2 decreased significantly in melanoma cells as soon as one day in the medium lacking riboflavin. These data suggest diverse ways how PQC can detect and degrade individual cofactor-free flavoproteins. Different mechanism can be operational even for the same flavoprotein. For example, it has been shown, that mutant and wild-type apo-NQO1 in vitro can be directly recruited to and degraded by the 20S proteasome [58].

## 7. POR

Cytochrome P450 oxidoreductase (POR) is an example of a flavoprotein that accommodates two flavin cofactors in its structure. POR is associated with FMN at the N-terminal flavodoxin domain and with FAD at the C-terminal ferredoxin reductase domain. The two cofactors orient toward each other in an alignment necessary for efficient electron transfer, their isoalloxazine rings being only 4 Å apart at the closest [59]. Electrons are brought to POR by NADPH and are transferred in the form of a hydride ion to FAD, passed from FAD to FMN, which then finally transfers them to one of many partner cytochromes P450 in the endoplasmic reticulum. The arrangement between flavins is permissive for the efficient inter-protein electron transfer; however, FMN needs to re-accommodate to donate electrons further. To this end, a more open conformation of the flavodoxin domain is required to position the reduced FMN in the vicinity to the heme-binding site in the cytochrome [60]. A single 21 amino acid-long segment close to the N terminus of the flavoprotein spans the endoplasmic membrane and anchors the reductase in the compartment where the partner cytochromes are localized.

Opposite to the anti-ferroptotic roles of other flavoprotein as discussed above, POR was recently shown to possess a pro-ferroptotic activity [21,61]. Two CRISPR-Cas9-based screens were set up to screen for ferroptosis suppression, whereby intrinsically sensitive clear-cell renal carcinoma and inherently resistant melanoma cell lines were used. The resistant cells were rendered ferroptosis-sensitive by supplementation with ω-6 and ω-3 PUFAs, and ML210 was used to trigger ferroptotic cell death. Inactivation of POR turned out to prevent from ferroptosis in both screens. Mechanistically, lipidome analysis revealed the involvement of POR in the lipid peroxidation [21]. The authors suggested that POR can facilitate lipid peroxidation by accelerating the cycling between Fe(II) and Fe(III) in the heme of partner cytochrome P450. At the same time, the participation of other electron acceptors cannot be excluded, because POR can donate electrons to a number of other redox partners, such as cytochrome b5, squalene monooxygenase and heme oxygenase [62]. The discovery is an important advance towards comprehending the diversity of pro-ferroptotic mechanisms. Previously, primarily the arachidonate lipoxygenases (LOXs) were thought to be responsible for lipid peroxidation behind ferroptosis induction [18]. However, lack of LOX expression in some ferroptosis-sensitive cells lines suggested additional lipid damage pathways. The POR-dependent lipid peroxidation offers a possible explanation of this discrepancy. In support, POR was also a hit in a genome-wide ferroptosis screen in the pancreatic carcinoma cell line KP-4 [61].

Because of its many client enzymes, POR defects are expected to result in complex metabolic disturbances, including manifold defects of steroidogenesis. This assumption proved true by the first molecular description of the clinical POR deficiencies that had manifested in the combined insufficiency of 17α-hydroxylase and 21-hydroxylase [63]. Many more mutations in POR have been identified since then. POR deficiency is now classified as a separate form of congenital adrenal hyperplasia independent of Antley–Bixler Syndrome [64]. It affects the biosynthesis of steroids in both the adrenal gland and the gonads. In addition to clinically manifest variants, there are some noticeable polymorphisms in POR not yet linked with disease. Conceivably, they might become relevant phenotypically during environmental variations. One example is the variant A503V, with an average allele frequency of 30%. The high frequency of A503V can also explain its increased detection in tumor samples. In contrast, the S35L variant is frequent in tumors, but not in healthy tissue of general population. The mutation is localized in the transmembrane segment of POR and thus can disturb its localization and function during tumorigenesis.

Can the availability of the flavin cofactors affect the structural stability and enzymatic performance of POR? We are not aware of the respective data regarding the wild-type enzyme. In the case of disease-causing mutations, the localization of the structural defect determines the remaining association of the variant protein with the cofactor. For example, V492E contains stoichiometric amounts of FMN and almost completely lacks FAD, whereas R475H, next to the normal amount of FMN, still contains FAD at ca. 35% of the wild-type value [65]. Both mutations disrupt the interaction of POR with the FAD pyrophosphate; however, there are subtle, yet distinct differences in the destabilization of the mutant enzymes. As a consequence, R457H results in less strong, but more global destabilization, whereby the mutant POR populates three ensembles in solution, one of which cannot be reconstituted with FAD. In contrast, the V492E mutant can be reloaded with FAD fully. An interesting case of the mutant POR reactivation was provided by Nicolo et al. [66]. The authors suggested that free FMN can compensate for the loss of the protein-bound cofactor in some mutants; for example, in the Y181D and A287P variants. The exact mechanism of this effect remains to be determined, including the clarification whether NADPH passes electron to FMN directly or via FAD.

POR belongs to the so-called class II redox partners of cytochrome P450. This class is widely represented among eukaryotes. The bacterial class I redox partner system is composed of two separate components, an FAD-bound ferredoxin reductase and a 2Fe-S cluster-bound ferredoxin. The eukaryotic mitochondria contain a class I system. Because of mitochondrial localization, the involvement of the flavoprotein ferredoxin reductase in ferroptosis is less probable, similarly as in the case of the FSP1 paralog AIFM1.

## 8. Other Flavoproteins from the Ferroptosis Network

In addition to the four described flavoproteins, several more can be reasonably considered as members of the ferroptosis network in human cells. For example, the flavoprotein squalene monooxygenase (SQLE) is, next to HMG-CoA reductase (HMGCR), the rate-limiting step of the mevalonate pathway. The pathway is involved in the biosynthesis of the endogenous membrane resident antioxidant ubiquinone. As expected, inhibition of HMG-CoA reductase with statins enhanced FIN56-induced ferroptosis, and this effect was reversed by the supplementation of cell cultures with mevalonic acid [16]. In the same study, the inhibition of the flavoprotein SQLE with NB-598 enhanced ferroptosis. SQLE is downstream of HMGCR and of the branching point towards ubiquinone. The pro-ferroptotic activity of this flavoprotein must be subordinate or weaker than that of the upstream pathway to explain the opposite effect brought about by statins.

Among electron-receiving POR partners are cytochromes b5, the small heme-bound proteins localized at cellular membranes. A group of five human flavoproteins is specialized in the reduction in cytochrome b5 and in this aspect they can be considered as functional POR analogues. The cytochrome b5 reductases (CYB5Rs) are membrane proteins found predominantly at the endoplasmic reticulum and the outer mitochondrial membrane. It was shown that CYB5R purified from liver plasma membrane can reduce CoQ in reconstituted liposomes [67]. In the presence of CoQ and NADH, the enzyme prevented liposomal peroxidation by parinaric acid. This anti-ferroptotic activity is noteworthy, given the functional similarity of CYB5R with the pro-ferroptotic POR [21].

Thioredoxin reductases (TXNRDs) have been mentioned in the section on GSR, yet these flavoproteins deserve additional discussion because they constitute another major thiol-dependent antioxidant system in mammalian cells. Human TXNRD1 is mainly, but not exclusively, found in the cytosol. The localization of its “v3” splice variant at the plasma membrane [68] and the capacity of the enzyme to reduce lipid hydroperoxides [69] are relevant when considering the possible roles of thioredoxin reductase in ferroptosis processes. In TXNRDs, the electrons are transferred from NADPH to FAD, then to N-terminal redox-active dithiol motifs, subsequently to the selenylsulfide of the other subunit of the complex, and finally to disulfide substrate of the reductase, the thioredoxin 1 [70]. Crosstalk between thioredoxin and glutathione systems is possible. For example, physiological concentrations of glutathione, NADPH, and GSR reduced thioredoxin in vitro and this reaction was strongly stimulated by glutaredoxin 1 [71]. Reciprocally, purified thioredoxins reduced glutathione in the presence of TXNRD and NADPH in vitro and thioredoxin system was able to compensate the lack of glutathione reductase [29]. Recently, the efforts to identify the mechanism of ferroptosis induction by ferroptocide revealed thioredoxin as its target, experimentally supporting the anti-ferroptotic role of the thioredoxin system [72].

Mitochondria are one of the major cellular sources of reactive oxygen species. Interestingly, it turned out that mitochondria have a key role in cysteine deprivation-induced ferroptosis but not in GPX4 inhibition-induced ferroptosis [73]. The authors showed that the inhibition of mitochondrial TCA cycle and the electron transfer chain suppress lipid peroxidation and ferroptosis. The relationship between TCA cycle and ferroptosis has been underlined by a recent study which found the flavoprotein dihydrolipoamide dehydrogenase (DLD) responsible for the cysteine deprivation-induced ferroptosis in head and neck cancer cell lines [17]. DLD is a component of the α-ketoglutarate dehydrogenase (KGDH), the complex decarboxylating α-ketoglutarate during TCA cycle. The addition of a-ketoglutarate to cell cultures enhances ferroptosis during cysteine deprivation even in the absence of glutamine. The ferroptotic role of DLD is interesting from a therapeutic perspective. It has been shown that riboflavin supplementation can rescue DLD mutants that cause mitochondrial myopathy [74]. Whether the absence of the riboflavin destabilizes wild-type DLD and how much this destabilization affects ferroptosis induction in tumor cells remains to be determined [75].

It cannot be excluded that more flavoproteins will be assigned the ferroptotic function. In silico approaches might help discovering new members and better understanding the function of the already known members of the ferroptosis-related flavoproteome. The analyses of shared structural features seems to be a promising strategy to this end [5].

## 9. Conclusions

Since 2012, when it was proposed, the concept of ferroptosis as a distinct form of cell death driven by the iron-dependent lipid peroxidation underwent impressive development. The mechanistic details of ferroptosis and its contribution to redox biology and disease have been rapidly clarified [10,76,77]. The involvement of ferroptotic pathways in diverse pathologies, such as tumors, neurodegeneration, stroke, ischemia-reperfusion injury among others, in combination with the successful development of chemical tools to manipulate lipid peroxidation, justifies the expectations of a strong clinical impact. However, a number of mechanistic questions and therapeutic challenges remains [77]. One of the remaining issues is the comprehensive understanding of the cellular states determining susceptibility to ferroptosis. Because flavoproteins are numerously involved in the molecular ferroptosis network, the flavoproteome function must determine these states, at least partially. Accordingly, this survey was motivated by the conviction that a detailed understanding of the human flavoproteome in the context of the ferroptosis network can be rewarding. Flavoproteins are enzymes and, as such, represent classical targets for drug development. The mostly non-covalent association with flavin cofactors offers a means to control the structural stability and thus the function of the flavoproteins. The key issue that needs to be understood in the future is the balance between pro-ferroptotic and anti-ferroptotic roles of flavoproteins and how this equilibrium can be shifted in either direction according to the therapeutic needs.

## Figures and Tables

**Table 1:** Human flavoproteins discussed in this review.

Gene	Flavoprotein	Flavin
AIFM1	Apoptosis inducing factor, mitochondria associated 1 ^1^	FAD
AIFM2	Ferroptosis suppressor protein 1 ^1^	6-hydroxy-FAD
CYB5R1-5	Cytochrome B5 reductases 1-5 ^1^	FAD
DLD	Dihydrolipoamide dehydrogenase	FAD
GSR	Glutathione-disulfide reductase	FAD
NQO1	NAD(P)H Quinone dehydrogenase 1	FAD
NQO2	Ribosyldihydronicotinamide dehydrogenase [quinone]	FAD
POR	Cytochrome P450 oxidoreductase ^1^	FMN + FAD
SQLE	Squalene monooxygenase ^1^	FAD
TXNRD1	Thioredoxin reductase 1	FAD

^1^ Membrane association has been well-established.

## Abbreviations

AIF	apoptosis-inducing factor
CHIP	C terminus of HSC70-interacting protein
CYB5R	cytochrome b5 reductases
DLD	dihydrolipoamide dehydrogenase
FAD	flavin adenine dinucleotide
FMN	flavin mononucleotide
FSP1	ferroptosis suppressor protein 1
GPX4	glutathione peroxidase 4
GSR	glutathione reductase
HMGCR	HMG-CoA reductase
LOX	lipoxygenase
NQO	NAD(P)H:quinone oxidoreductase
POR	cytochrome P450 oxidoreductase
PQC	protein quality control
PUFA	polyunsaturated fatty acids
redox	reduction and oxidation
QXOS	quiescin sulfhydryl oxidase
SQLE	squalene monooxygenase
TXNRD	thioredoxin reductase
